# Optisample^TM^: Open web-based application to optimize sampling strategies for active surveillance activities at the herd level illustrated using Porcine Respiratory Reproductive Syndrome (PRRS)

**DOI:** 10.1371/journal.pone.0176863

**Published:** 2017-07-18

**Authors:** Anna Alba, Robert E. Morrison, Ann Cheeran, Albert Rovira, Julio Alvarez, Andres M. Perez

**Affiliations:** 1 Department of Veterinary Population Medicine, College of Veterinary Medicine, University of Minnesota, St. Paul, MN, United States of America; 2 Veterinary Diagnostic Laboratory, University of Minnesota, St. Paul, MN, United States of America; Universitat de Lleida, SPAIN

## Abstract

Porcine reproductive and respiratory syndrome virus (PRRSv) infection causes a devastating economic impact to the swine industry. Active surveillance is routinely conducted in many swine herds to demonstrate freedom from PRRSv infection. The design of efficient active surveillance sampling schemes is challenging because optimum surveillance strategies may differ depending on infection status, herd structure, management, or resources for conducting sampling. Here, we present an open web-based application, named ‘Optisample^TM^’, designed to optimize herd sampling strategies to substantiate freedom of infection considering also costs of testing. In addition to herd size, expected prevalence, test sensitivity, and desired level of confidence, the model takes into account the presumed risk of pathogen introduction between samples, the structure of the herd, and the process to select the samples over time. We illustrate the functionality and capacity of ‘Optisample^TM^’ through its application to active surveillance of PRRSv in hypothetical swine herds under disparate epidemiological situations. Diverse sampling schemes were simulated and compared for each herd to identify effective strategies at low costs. The model results show that to demonstrate freedom from disease, it is important to consider both the epidemiological situation of the herd and the sample selected. The approach illustrated here for PRRSv may be easily extended to other animal disease surveillance systems using the web-based application available at http://stemma.ahc.umn.edu/optisample.

## Introduction

Since its first recognition in 1987, porcine reproductive and respiratory syndrome virus (PRRSv) infection has been described in many countries worldwide. The disease has a high economic impact on the swine industry of infected countries, including North America, by causing an increase in mortality, decrease in growth performance in growing pigs, abortions, stillbirths and premature farrowing in breeding herds. Moreover, PRRSv infection is also associated with respiratory disease, pyrexia, and anorexia at all ages [1]. Some studies have described diverse strategies for the PRRSv control and elimination at herd and regional levels. PRRSv elimination programs are based on strict internal biosecurity in conjuction with herd closure until the virus is thought to have been eliminated [2–4].

The confidence level in having eliminated the virus from the herd is often based on a combination of absence of clinical signs and negative lab results from consecutive samplings conducted in the population [5, 6]. After having eliminated the virus from the herd, active surveillance is routinely carried out in many swine herds to demonstrate ongoing freedom from PRRSv infection [7, 8]. Identification of cost-effective sampling strategies is crucial to implement effective and sustainable surveillance systems. However, specifying a suitable sampling strategy is not always straightforward as surveillance schemes are governed by a number of parameters, such as the minimal detectable prevalence, the herd size, and the accuracy of the diagnostic tests. Furthermore, the optimum strategy may differ between farms depending on their infectious status, their structure and management, the epidemiological situation, and the resources available. As a result, to enhance active surveillance, practical methods to identify efficient sampling strategies taking into account the situation of the herd and costs are required.

The aim of the work is to illustrate the use of ‘Optisample^TM^’, a flexible and accessible modeling tool for stakeholders and veterinarians that computes and compares the probability of being free of infection (*PFree*) and costs of different sampling strategies considering the epidemiological situation of each herd and the sampling selection process. ‘Optisample^TM^’ is an expanded version of the models proposed by Cannon (2002) [9] and Martin (2007, 2008) [10–11] to substantiate freedom from disease at the herd level. ‘Optisample^TM^’ may be easily applicable to other diseases and populations to inform decisions related with surveillance, and ultimately prevention and control of animal diseases.

## Materials and methods

### Inputs and outputs of ‘Optisample^TM^’

The model formulation and parameterization intends to estimate the probability of freedom from disease by taking into account the sampling strategy as well as characteristics of herd demography and disease epidemiology. Key parameters are estimated based on observational data regarding disease occurrence in the past. Demographic and epidemiologic features are specified via the herd size (*N*), the start of the observation period (*hd*), the end of the observation period (*cd*), the number of outbreaks occurred during the period of observation (*n*_*ou*_), the expected duration of pathogen persistence in the herd (*p*_*p*_), the time span between two outbreaks occurred (*f*_*ou*_) and the correlation between successive sampled groups for the pathogen prevalence (*ICC*_*bt*_). The sampling strategy was included via the expected prevalence to detect (P*), the frequency of testing (*f*_*t*_), the number of tested samples (*n*_*t*_), and the sensitivity (*se*_*test*_), and the cost (*Price*_*test*_) of a laboratory tests.

Noteworthy, the test specificity here is assumed to be perfect (i.e. 100%) because, in the event of positive result, it is expected that an exhaustive epidemiological investigation would take place at herd and sufficient samples would be collected and tested to rule out false positive results.

If all tests are negative, ‘Optisample^TM^’ provides as outputs the *PFree* and the overall cost of testing (*Cost*_*t*_). Based on the representativeness of the sample of the herd and the pathogen distribution within the herd, the model estimates the *PFree* for each herd simulating two scenarios (named *S* and *D*). In scenario *S* is assumed that the pathogen is homogeneously distributed and a representative random sample is always selected from the herd over time. In contrast, in scenario *D*, it is assumed that the pathogen distribution is heterogeneous among different sub-units of the herd and the sampled sub-units vary over time. This second scenario would be explained by demographic structure, biosecurity measures, management of the farm and logistics of sample collection. An example of this situation would be in sow herds, where, to determine the herd status of PRRSv, the producers often conduct samplings only in piglets that are to be weaned. In these cases each sampling is conducted in different sub-units of animals each time. The *PFree* is estimated after each sampling *t* for both scenarios *S* and *D* (*PFree*_*S*,*t*_ *PFree*_*D*,*t*_) and over a time frame of 12 days, 12 weeks or 12 months. The probability of freedom over this time frame is approximated by computing the area under the curve, which is referred to as *AUC*_*S*_ or *AUC*_*D*_ according to the corresponding simulated scenario.

Parameters used in ‘Optisample^TM^’ are shown in Table 1.

**Table 1:** Summary of the inputs set by the user and outputs of ‘Optisample^TM^’.

Parameter	Notation	Data type	Range
Inputs			
Demographic and epidemiologic traits of the herd			
Herd size	*N*	Integer	0 - ∞
Start of the observation period	*hd*	Date (yyyy-mm-dd)	No limits
End of the observation period	*cd*	Date (yyyy-mm-dd)	Automatically determined
Number of outbreaks that occurred during the period of observation	*n*_*ou*_	Integer	0 - ∞
Expected duration of pathogen persistence in a herd in the event of an outbreak (in days)	*p*_*p*_	Unif (min, max)	1–365
Time spam between two outbreaks occurred (in years)	*f*_*ou*_	Integers: min, max	1–15
Correlation between successive sampled groups for the pathogen prevalence	*ICC*_*bt*_	Unif (min, max)	0–1
Sampling strategy			
Frequency of testing	*f*_*t*_	Factor (3 levels)	Daily, weekly, monthly
Minimum prevalence to detect	P*	Fixed proportion	0–1
Sample size of consecutive samplings	*n*_*t*_	Sequence of 12 integers	0–300
Diagnostic test sensitivity	*se*_*test*_	Pert (min, mode, max)	0–1
Price for unit lab test	*Price*_*test*_	Numeric value	0 –∞
Outputs			
Pr. free of infection after sampling *t* for scenario *S*	*PFree*_*S*,*t*_	min–md–max	0–1
Pr. free of infection after sampling *t* for scenario *D*	*PFree*_*D*,*t*_	min–md–max	0–1
Pr. free of infection over all period for scenario *S*	*AUC*_*S*_	min–md–max	0–1
Pr. free of infection over all period for scenario *D*	*AUC*_*D*_	min–md–max	0–1
Cost of testing	*Cost*_*test*_	Numeric value	0–9999999

scenario *S*: assuming homogeneous pathogen distribution and collecting random samples from all herd over time

scenario *D*: taking into account heterogeneous pathogen distribution and collecting samples from different animal sub-units over time

### Modelling process

The time frame assessed by the model (i.e. 12 days, 12 weeks or 12 months) depends on the frequency of consecutive testings (*f*_*t*_) set by the user (daily, weekly or monthly). The model automatically scales all the inputs in days, weeks or months according to *f*_*t*_ to compute all the outputs.

The modeling process comprises different steps.

1. To estimate the probability that the herd is infected before conducting any sampling (*PI*_*t = 0*_) the the model makes use of four inputs. These inputs are (1) start of the observation period (*hd*), (2) end of the observation period (*cd*), (3) number of outbreaks that occurred during the period of observation (*n*_*ou*_), and (4) expected duration of pathogen persistence at the herd in the event of outbreak (*p*_*p*_). Here the value of *p*_*p*_ is defined using a continuous uniform distribution [12] with minimun and maximum expected duration values. To describe the uncertainty and variability of *PI*_*t = 0*_ the model computed its value using a Beta distribution with parameters α and β [13] automatically derived from *hd*, *cd*, *n*_*ou*_ and *p*_*p*_ following the expression:

$${PI}_{t=0}=Beta(\propto =n_{ou}*p_{p}+1,\;\beta =(cd-hd)–(n_{ou}*p_{p})+1)$$ (1)

where *n*_*ou*_ * *p*_*p*_ correspond to the period of time in which the pathogen may persist in the herd; and (*cd* − *hd*)−(*n*_*ou*_ * *p*_*p*_) correspond to the total period of time with available information during which there is no pathogen persistence.

2. At time *t* = 1 a first sampling is conducted on a number of animals (*n*_*1*_) using a given diagnostic test. The probability of detecting at least one infected animal if the herd is infected (*Se*_*t = 1*_) is estimated considering *n*_*1*_, a minimum proportion of infected animals within the herd that we would expect if the disease was present (P*), the size of the herd (*N*), and the sensitivity of the diagnostic test (*se*_*test*_). The value of P* is included as a fixed value that ranges between 0 and 1 and is set by the user based on the market-requirements or accreditation purposes. The *se*_*test*_ is expressed as a Pert distribution [14] with possible values ranging between 0 and 1.

$${se}_{test}=Pert(min,most\;likely,max,ë=4)$$ (2)

The values of the *se*_*test*_ may be determined based on the information provided by the veterinary diagnostic laboratory that processes the samples or based on available scientific references. Here, the user could set the 2.5th and 97.5th quantiles as proxy measurements for the boundary parameters if the values of *se*_*test*_ are expressed as 95% confidence interval.

The *Se*_*t = 1*_ is calculated using a hypergeometric approximation based on the approach proposed by Cameron and Baldock (1998) [15,16]. The *Se*_*t = 1*_ is expressed as:

$${Se}_{t=1}=1-{\left(1-{se}_{test}*\frac{n_{1}}{N}\right)}^{N*P^{*}}$$ (3)

3. If all the samples of *t* = 1tested negative, the model estimates the *PFree*_*t = 1*_ by simulating the scenarios *S* (*PFree*_*S*,*t = 1*_) and *D* (*PFree*_*D*,*t = 1*_). To assess the influence of selecting different sub-units over time, ‘Optisample^TM^’includes a parameter that represents the correlation between successive sampled groups for the pathogen prevalence (*ICC*_*bt*_). In scenario *S*, where the pathogen is homogeneously distributed throughout the herd, and the sampling is conducted over time in a unique and representative group of animals of the whole herd, the value of *ICC*_*bt*_ is equal to 1. Thus, the *PFree*_*S*,*t*_ estimated from a specific sampling can be directly extrapolated to the rest of the herd. Here, the *PFree*_*S*, *t = 1*_ is computed using a Bayesian inference approach that considers the *PI*_*t = 0*_ and the *Se*_*t = 1*_ [17] as follows:

$${PFree}_{S,t=1}=\frac{1-{PI}_{t=0}}{1-{Se}_{t=1}*{PI}_{t=0}}$$ (4)

In the scenario *D* the spread of infection within the herd differs among animal groups. The sub-units usually are interrelated, but are not exactly the same in terms of pathogen distribution. In this scenario, the estimates can only be partially extrapolated to the successive groups according to the value of *ICC*_*bt*_ defined as a continuous uniform distribution [12] that can take values between 0 and 1 considering the structure and management of the herd. In these cases the *PFree*_*D*,*t = 1*_ in successive groups or sub-units of the same herd depends on *ICC*_*bt*_ and is computed as:

$${PFree}_{D,t=1}=\frac{1-{PI}_{t=0}}{1-{Se}_{t=1}*{PI}_{t=0}}*{ICC}_{bt}$$ (5)

4. Once *PFree*_*S*,*t = 1*_ and *PFree*_*D*,*t = 1*_ are estimated, the model calculates the probability of having overlooked the disease in each respective scenario *S* and *D* (named *PI*_*S*,*t =* 1_ and *PI*_*D*,*t =* 1_) as:

$$\begin{array}{l} \;{PI}_{S,t=1}=1-{PFree}_{S,t=1}\\ \;\mathrm{and}\\ {PI}_{D,t=1}=1-{PFree}_{D,t=1} \end{array}$$ (6)

5. However, there also exists the possibility of pathogen incursion between consecutive samplings (*PI*_*bt*_). This value is highly variable, uncertain and mainly depends on trade movements, biosecurity measures, proximity to other infected farms and environmental viability. In this first version of the model, to facilitate the programming, computing and a better understanding of the influence of *PI*_*bt*_ on the results, the parameter is assumed as constant over time. The value of *PI*_*bt*_ is automatically derived from historical data using the minimum and maximum time span between outbreaks occurred in the herd (named *f*_*o(min)*_ and *f*_*o(max)*_) and the minimum and maximum periods of time in which the pathogen may persist in the herd (named *p*_*p(min)*_ and *p*_*p(max)*_). Here, in the event of no data, the user can set the minimum and the maximum values considered by the model. Here, the application computed the value of *PI*_*bt*_ as a Pert distribution [14] following the formula:

$${PI}_{bt}=Pert\left(min=\frac{p_{p(min)}}{f_{o(max)}},most\;likely=mean(\frac{p_{p(min)}}{f_{o(max)}},\frac{p_{p(max)}}{f_{o(min)}}),max=\frac{p_{p(max)}}{f_{o(min)}},\lambda =\right)$$ (7)

6. From the *PI*_*bt*_ and the respective values of *PI*_*S*,*t = 1*_ and *PI*_*D*,*t = 1*,_ the model computes for each scenario the overall probability that the herd is infected before the second sampling (*PrItot*_*S*,*t = 1*_ and *PItot*_*D*,*t = 1*_) [17] as follows:

$$\begin{array}{c} {PItot}_{S,t=1}={PI}_{bt}+{PI}_{S,t=1}-{PI}_{bt}*{PI}_{S,t=1}\\ \mathrm{and}\\ {PItot}_{D,t=1}={PI}_{bt}+{PI}_{D,t=1}-{PI}_{bt}*{PI}_{D,t=1} \end{array}$$ (8)

7. For each consecutive sampling *t*, the model develops an analogous process to the previous calculations (steps 2–6) to compute the values of *Se*_*t*_, *PFree*_*S*,*t*_, *PFree*_*D*,*t*_, *PI*_*S*,*t*_, *PI*_*D*,*t*_, *PItot*_*S*,*t*_ and *PItot*_*D*,*t*_ where *t* varies from 2 to 12.

8. The previous steps estimate the *PFree*_*S*,*t*_ and *PFree*_*D*,*t*_ after each sampling *t*. The area under the curve (*AUC*) is computed over all sampling events to estimate the overall probability of being free of infection. Here, the *AUC* is an integrated metric of the confidence of disease freedom for all the periods. Its computation used the sum of consecutive values of the respective *PFree*_*t*_ obtained from all consecutive samplings in each scenario based on the trapezoidal rule [18] as follows:

$$AUC=\Delta t\left(\frac{{PFree}_{t=1}}{2}+{PFree}_{t=2}+{PFree}_{t=3}+\cdots +\frac{{PFree}_{t=12}}{2}\right)$$ (9)

Where Δ*t* represents the elapsed time between consecutive samplings.

The *AUC* value ranges between 0 and 1 and indicates the probability that the herd was free from the infection throughout the assessed period, being 1 if *PFree* = 100%, and 0 if *PFree* = 0%. Depending on the scenario *S* or *D*, *AUC* is denoted as *AUC*_*S*_ (for homogeneous pathogen distribution and random sampling over time) or *AUC*_*D*_ (for heterogeneous pathogen distribution and sampled sub-units varying over time). The *AUC* is represented as minimum, median and maximum values taking into account the ranges for the inputs previously set into the model.

10. Finally the model computes the cost of testing (*Cost*_*test*_). The model sums all the samples tested over time and multiplies this value by a given cost of each individual test (*Price*_*test*_) provided by the user.

$${Cost}_{test}={Price}_{test}\;\sum_{t=1}^{t=12}n_{t}$$ (10)

### Visualization procedure

**Fig 1:**
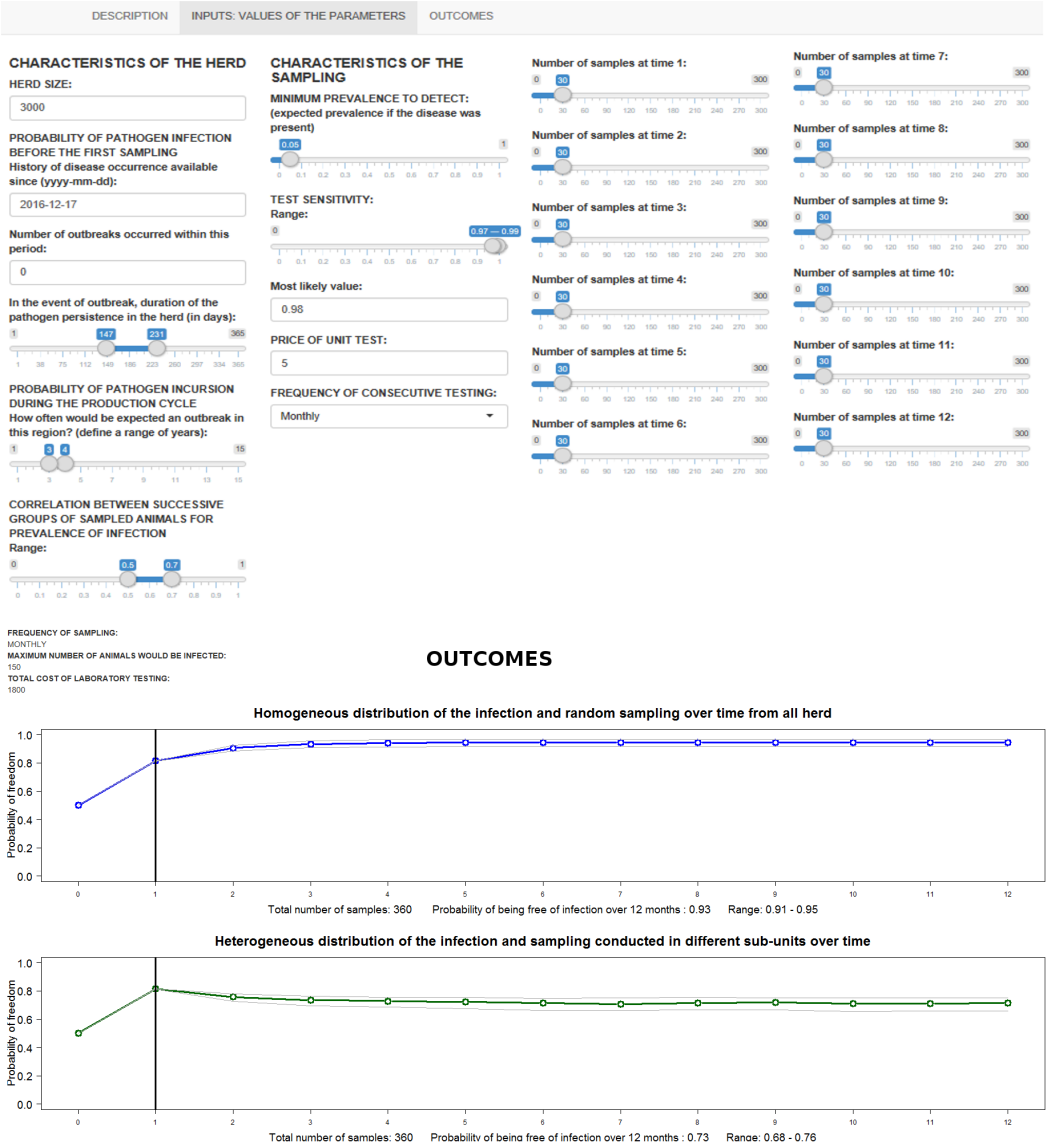
Layout of ‘Optisample^TM^ for the input values and outcomes.

‘Optisample^TM^’ is freely accessible at http://stemma.ahc.umn.edu/optisample. The layout of this web application has been displayed in three parts. The first part includes a basic explanation of the operation modeling. The second part consists of a panel of inputs in which the user sets the values of each parameter. Finally, the third part shows the outcomes represented in two plots indicating *PFree*_*S*,*t*_, *AUC*_*S*_, *PFree*_*D*,*t*_, *AUC*_*D*_ *and Cost*_*test*_. The second and the third part of the layout of Optisample^TM^’ are shown in Fig 1.

### Development environment

‘OptiSampleTM’ was developed using the statistical R software [19] and Rstudio [20] as integrated environment of R. The package ‘shiny’was used to build the interactive web application [21]. The package’mc2d’ was used to compute the pert distributions of the *se*_*test*_ and the *PI*_*bt*_ [14] and the package ‘RSurveillance’ was used to calculate the *Se*_*t*_ using the hypergeometric approximation assuming a known population size [16].

### Simulation of scenarios

To illustrate the functionality of ‘Optisample^TM^’, we estimated and compared the *PFree* of PRRSv using different schemes in three hypothetical swine herds located in regions with disparate epidemiological situations and infection status. The status of these herds was determined considering the PRRSv shedding and exposure according to the standardized terminology defined by the American Association of Swine Veterinarians [22]. In all scenarios we aimed at detecting a hypothetical design prevalence of 5%, a common threshold used to eliminate PRRSv from the herds by herd closure [23]. These three herds had some features in common such as herd size, expected duration of the pathogen persistence in the herd in the event of an outbreak and values of correlation between successive sampled groups for the pathogen prevalence. The herd size was 3,000 animals. The minimum and maximum values of pathogen persistence for PRRSv based on the previous studies were set between 147 and 231 days [24]. The correlation between successive sampled groups for the pathogen prevalence to simulate the scenario *D* ranged between 0.5 and 0.7.

Herd A was a multiplier herd with very low incidence of PRRSv (i.e. one outbreak every 5 or 6 years). This herd had a negative infection status (IV). The disease status in this herd had been followed for the last 5 years and no outbreaks had occurred during this period. No pigs had been introduced recently, the level of biosecurity was high, and the number of pig movements into other farms was relatively small. The objective here would be to demonstrate that herd A was free from PRRSv with a 95% confidence level testing individual sera with a commercial PRRSv antibody ELISA kit with a sensitivity of 98% (97%–99%) [25–26]. We assumed a price of USD 5 per serological test.

Herd B was a commercial herd with negative infection status (IV) with a high incidence of PRRSv (i.e. one outbreak every 2 to 3 years). The farm had introduced new pigs. The disease status of the herd of origin was unknown, and due to the lack of information the model set the *PI*_*t = 0*_ automatically to 0.5. The aim here would be to demostrate that herd B was free of PRRSv infection with a 95% confidence level using the same commercial PRRSv antibody ELISA kit used for herd A.

Herd C was a commercial herd with medium incidence (i.e. one outbreak every 3 to 4 years). This herd was classified as positive stable undergoing elimination according to the RT-qPCR positive at weaning (II-B). Here the objective would be to assure that the infection had been eliminated. Sera samples were tested using a PRRSv RT-qPCR with a sensitivity of 98% (97%–99%) as described elsewhere [27]. In this case there was evidence that the herd had been recently infected, and thus for the *PrI*_*t = 0*_ the user could set hd to the initial date of the outbreak (here, as example, we set the date of two months ago) and a value of 1 as number of outbreaks occurred since this date. We assumed a hypothetical cost of USD 10 per molecular test.

Three sampling schemes conducted over the course of a year were assessed for each of the herds. In sampling scheme I 30 samples were collected per month in each herd. In sampling scheme II 50 samples were collected per month in each herd. In sampling scheme III the strategy varied in each herd (i.e. IIIa, IIIb and IIIc) to achieve a ~ 95% probability of being free of infection at lower cost in the scenario *S* (i.e. homogeneous distribution of the infection in the herd) (Table 2).

**Table 2:** Inputs and outputs for the proposed scenarios.

Inputs									
Notation	Herd A			Herd B			Herd C		
*N*	3000			3000			3000		
*hd*	Date 5 years ago			Current date (0 months)			Date 2 months ago		
*cd*	Current date			Current date			Current date		
*n*_*ou*_	0			Unknown (n.d.)			1		
*p*_*p*_	Unif (147, 231)			Unif (147, 231)			Unif (147, 231)		
	min: 147, max: 231			min: 147, max: 231			min: 147, max: 231		
*f*_*ou*_	min: 5, max: 6			min 2, max: 3			min:3, max:4		
*ICC*_*bt*_	Unif (.5, .7)			Unif (.5, .7)			Unif (.5, .7)		
*f*_*t*_	monthly			monthly			monthly		
P *	.05			.05			.05		
*se*_*test*_	Pert(.97, .98, .99)			Pert(.97, .98, .99)			Pert(.97, .98, .99)		
*Price*_*test*_	5			5			10		
Sampling									
*Scheme*	I	II	IIIa	I	II	IIIb	I	II	IIIc
	30 samples monthly	50 samples monthly	50 samples bimonthly	30 samples monthly	50 samples monthly	60 at t = 1 and 40 monthly	30 samples monthly	50 samples monthly	90 at t = 1 and 35 monthly
*Tota*_*nt*_	360	600	300	360	600	500	360	600	475
Outputs									
*AUC*_*S*_	.96-.97-.98	.98-.99-.99	.91-.93-.95	.85-.89-.94	.95-.97-.98	.92-.96.-97	.78-.82-.84	.92-.93-.94	.93-.95-.96
*AUC*_*D*_	.76-.78-.8	.92-.93-.93	.61-.63-.66	.59-.68–74	.87-.9-.92	.77-.83-.86	.52-.58-.61	.85-.86-.87	.76-.79-.81
*Cost*_*test*_	1800	3000	1500	1800	3000	2500	3600	6000	5750

## Results

**Fig 2:**
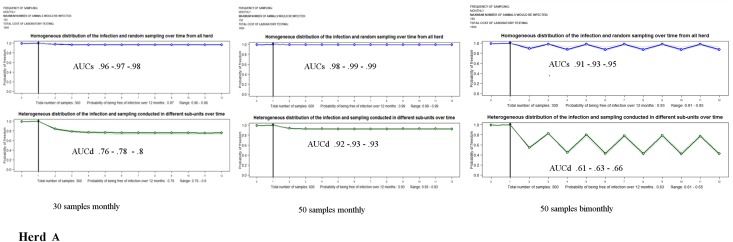
Monthly probabilities of freedom (blue and green) and range bands (grey) for herd A (a multiplier herd with a low initial probability of PRRSv infection and low risk between consecutive samplings).

**Fig 3:**
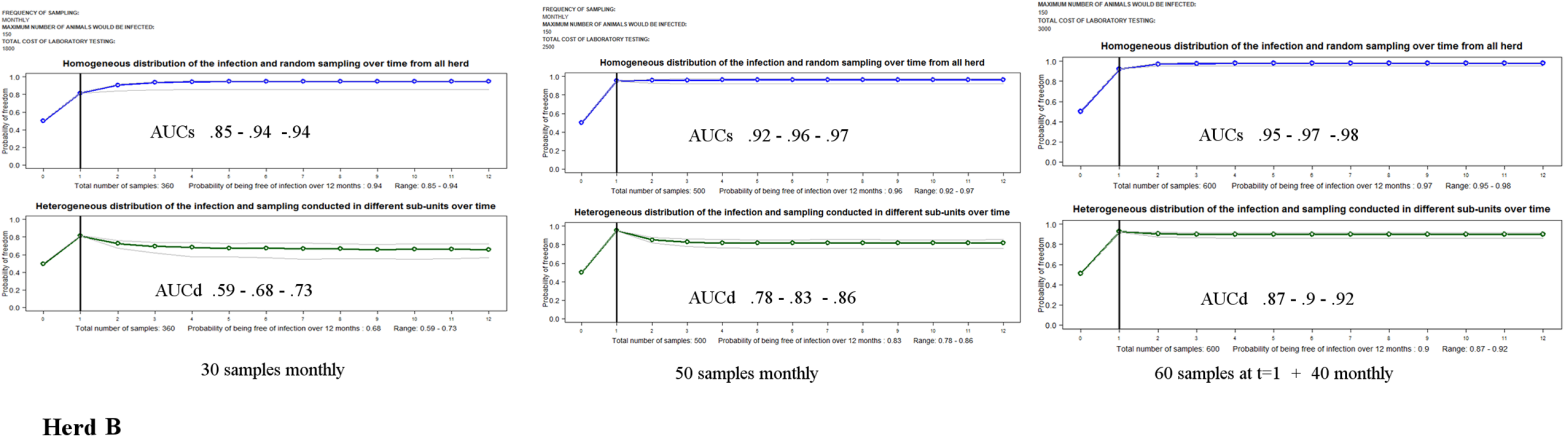
Monthly probabilities of freedom (blue and green) and range bands (grey) for herd B (a commercial herd with unknown probability of infection initially and a high probability of infection between consecutive samplings).

**Fig 4:**
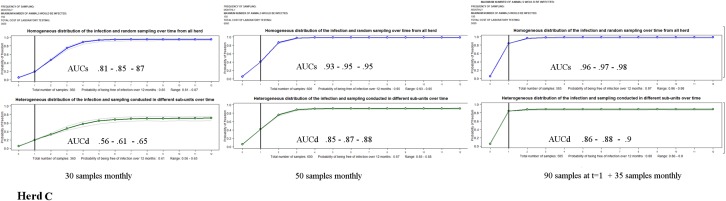
Monthly probabilities of freedom (blue and green) and range bands (grey) for herd C (a commercial positive stable pig herd undergoing elimination with a medium probability of infection between consecutive samplings).

The probability of being free from PRRSv infection for the herds A, B and C after conducting consecutive sampling over one year with the costs of testing are shown in Table 2 and plotted in Figs 2–4.

The comparison of outcomes of *AUCs s*ampling 30 animals by month illustrated that the confidence of being free from PRRSv over the entire period decreased when increasing the probability of being infected initially or between successive samplings, with median values of 0.97 for herd A, 0.89 for herd B, and 0.82 for herd C.

*AUC*_*D*_ results for herds A, B and C indicated a marked decrease of confidence if the pathogen was assumed to be heterogeneously distributed between sub-units in the herd. In these scenarios, to substantiate freedom from PRRSv, it would be necessary a substantial increase in the pressure of sampling, almost doubling the number of samples over time (see scheme II for herds A and B).

The results of herd C showed that, to demonstrate freedom from infection when the risk of being initially infected was high, it was necessary to substantially increase the sample size during the first samplings. For herd C, due to the cost of the molecular test, the final budget was higher than for herds A and B.

Plots depicted in Fig 2, Fig 3 and Fig 4 allowed comparing the monthly results computed over time for the different scenarios. These outputs showed how influential the initial probability of infection was to demonstrating freedom from infection over time. The impact was more evident in herd C. Furthermore, the patterns of the figures demonstrated the ability of substantiating disease freedom over time based on cumulative information obtained from previous sampling and how the probability of infection between consecutive sampling might impact these estimates. Here, the lines connecting the monthly estimates showed an increase in those months in which samplings were conducted and a decrease in those months in which there was no sampling (see scheme IIIa).

## Discussion

Prevention, control, and eradication of infectious animal diseases at herd level require access to up-to-date information on the infection status of the herd. Most of this information is often obtained from periodic samplings usually conducted following predetermined schemes. For example, for PRRSv, a relatively common practice in sow herds intending to demonstrate freedom from infection is to test serum samples from 30 weaned pigs. When no RT-qPCR positive results are obtained in four consecutive samplings, it is estimated with a 95% confidence that the PRRSv prevalence in the herd is below 10% and the herd is considered free from PRRSv infection. The outputs of our model demonstrate how this strategy is affected by the epidemiological situation of the herd; hence, a general strategy to demonstrate disease freedom may not serve equally well in different epidemiological situations. The modelling approach presented here allows to introduce inputs taking into account their variability and uncertainty, and to assess the influence of different determinants on the probability of freedom from infection.

The model illustrates the importance of checking the health status of animals at the arrival to maximize the likelihood that introduced animals are not infected. If the probability of being free at the start is low or there is no historical data available to determine *PI*_*t = 0*_ (here by default *PI*_*t = 0*_ = 0.5), the model shows that, to demonstrate freedom from PRRSv, we need to test a higher number of samples during the initial samplings, compared to other scenarios (herds C and B versus herd A). Moreover, depending on the initial infection status of the herd, the risks of disease introduction, the impact of the disease and the immediate aims to achieve, the sampling and testing protocol might be different. For example in herd A, which did not introduce pigs or had a small number of movements among herds, the risks of initial infection or between consecutive samples were low. In this herd the very early diagnosis was not as important as it was in herd B. On the other hand, if new animals had been introduced into the herd at t = 0 or between consecutive samplings, the probability of being infected initially or between consecutive samplings varied, influencing the distribution of the pathogen in herd. In contrast, in herd B, there were many animal movements, and we might be interested in detecting lower levels of prevalence than in herd A, and thus, the early detection of viraemic animals may be more critical. In this case, the use of a RT-qPCR test to detect viraemic animals at earlier stages, a selection of a lower P* and the increase of the sampling frequency could be more appropriate. In this sense, ‘Optisample^TM^’ might help to assess the probability of being free over time adjusting the hypothetical prevalence, test sensitivity and sampling frequency. Indeed, when the values of P* or *se*_*test*_ are lower, larger sample sizes are required to demonstrate that the herd is free from disease.

The probability of being free over time also depends on the risk of incursion between consecutive samplings, as demonstrated by the model outputs. Such risk varies according to biosecurity measures in place, the frequency of direct or indirect contacts with other infected herds, and the viability of PRRSv in the environment. When the risk of disease introduction between consecutive samplings is low, the previous negative outputs also provide cumulative information to substantiate that the herd is free from infection. As a result, the lag between samplings may be extended while maintaining a high confidence in disease freedom (see scheme IIIa for herd A). In contrast, when the probability of incursion between samplings is high, the probability of being free over time becomes low and the frequency of samples should not decrease (see schemes IIIa and IIIb).

‘Optisample^TM^’ also illustrates the importance of sample selection. To the best knowledge of the authors, previously available software [28, 29] to calculate sample size in order to detect infection assume that, in the event of infection, this will be homogeneously distributed across the herd. However, from our model outputs, it seems evident that, if the groups sampled are heterogeneous and different sub-units of animals are sampled over time, the confidence of disease freedom decreases dramatically. The value of *ICC*_*bt*_ may be challenging to estimate, given that this parameter depends on management and structure of each farm. Thus, to get plausible values for each case, we would require a specific model to assess the pathogen spread within each herd. Still, we believe the inclusion of this parameter demonstrates the importance of assessing the process of sample selection to substantiate freedom from disease.

As a limitation of the model, it is important to remark that in this initial version of Optisample^TM^, to facilitate programming, computation, and a better understanding of the process, the herd size and the risk of incursion between consecutive samplings are set as constant values throughout the entire study period. However, in those herds in which the herd size or the risk of pathogen incursion varies over the period of study, such assumption may lead to biased results, and thus the interpretation of outcomes may be misleading. A potential extension in future versions to improve the accuracy of outputs would be to estimate the risk of incursion between samplings according to season or other associated factors using available continuous information of each herd.

In summary, the work here illustrated a novel approach to enhance the design of active surveillance for PRRSv at herd level. Additionally, the approach here, including its principles and methods, may be easily extended to other surveillance contexts for a variety of species and animal diseases. This freely available application contributes to assessing the importance of the main factors affecting the probability of disease freedom at herd level, ultimately supporting management decisions to prevent and mitigate the impact of animal diseases on susceptible populations.

## References

[1] Zimmerman JJ, Yoon KJ, Wills RW, Swenson SL. General overview of PRRSV: a perspective from the United States. Vet Microbiol. 1997; 55(1): 187–196. 9220613 10.1016/s0378-1135(96)01330-2

[2] Corzo CA, Mondaca E, Wayne S, Torremorell M, Dee S, Davies P et al Control and elimination of porcine reproductive and respiratory syndrome virus. Virus Res. 2010; 154(1): 185–192. 20837071 10.1016/j.virusres.2010.08.016

[3] Lambert MÈ, Poljak Z, Arsenault J, D’Allaire S. Epidemiological investigations in regard to porcine reproductive and respiratory syndrome (PRRS) in Quebec, Canada. Part 1: Biosecurity practices and their geographical distribution in two areas of different swine density. Prev Vet Med. 2012; 104(1):74–83. 22243985 10.1016/j.prevetmed.2011.12.004

[4] Rowland RR, Morrison RB. Challenges and opportunities for the control and elimination and elimination of porcine reproductive and respiratory syndrome virus. Transbound Emerg Dis. 2012; 59(s1):55–9. 25471243 10.1111/j.1865-1682.2011.01306.x

[5] Stark KD, Hasler B. The value of information: Current challenges in surveillance implementation. Prev Vet Med. 2015; 122(1): 229–234. 26021437 10.1016/j.prevetmed.2015.05.002

[6] Holtkamp DJ, Yeske PE, Polson DD, Melody JL, Philips RC. A prospective study evaluating duration of swine breeding herd PRRS virus-free status and its relationship with measured risk. Prev Vet Med. 2010; 96(3):186–193. 20692057 10.1016/j.prevetmed.2010.06.016

[7] Amezcua R, Pearl DL., Friendship RM, 2013. Comparison of disease trends in the Ontario swine population using active practitioner-based surveillance and passive laboratory-based surveillance (2007–2009). Can Vet J, 2013 54:775–83. 24155479 PMC3711168

[8] Frössling J, Ågren EC, Eliasson-Selling L, Lewerin SS. Probability of freedom from disease after the first detection and eradication of PRRS in Sweden: scenario-tree modelling of the surveillance system. Prev Vet Med. 2009 1; 91(2):137–45. 19520445 10.1016/j.prevetmed.2009.05.012

[9] Cannon RM. Demonstrating disease freedom-combining confidence levels. Prev Vet Med. 2002; 52(3): 227–249. 11849719 10.1016/s0167-5877(01)00262-8

[10] Martin PAJ, Cameron AR, Barfod K, Sergeant ESG, Greiner M. Demonstrating freedom from disease using multiple complex data sources: Case study-Classical swine fever in Denmark. Prev Vet Med. 2007; 79(2): 98–115. 17239459 10.1016/j.prevetmed.2006.09.007

[11] Martin PAJ. Current value of historical and ongoing surveillance for disease freedom: Surveillance for bovine Johne’s disease in Western Australia. Prev Vet Med. 2008; 84(3): 291–309. 18243373 10.1016/j.prevetmed.2007.12.002

[12] Forbes C, Evans M, Hastings N, Peacock B. Statistical distributions John Wiley & Sons; 2011 3 21.

[13] Enøe C, Georgiadis MP, Johnson WO. Estimation of sensitivity and specificity of diagnostic tests and disease prevalence when the true disease state is unknown. Prev Vet Med. 2000;45(1):61–81. 10802334 10.1016/s0167-5877(00)00117-3

[14] Pouillot R, Delignette-Muller ML, Denis JB, Pouillot MR. Package ‘mc2d’. hist. 2016 Oct 11;1100:17.

[15] Cameron and Baldock. A new probability formula for surveys to substantiate freedom from disease. Prev Vet Med.1998, 34:1–17 9541947 10.1016/s0167-5877(97)00081-0

[16] Sergeant E. RSurveillance: Design and Analysis of Disease Surveillance Activities. R package version 0.1.0. 2014.

[17] Martin P.A.J., Cameron A.R. and Greiner M. Demonstrating freedom from disease using multiple complex data sources: 1: A new methodology based on scenario trees. Prev Vet Med. 2007; 79(2):71–97. 17224193 10.1016/j.prevetmed.2006.09.008

[18] Yeh, S.T., 2002. Using trapezoidal rule for the area under a curve calculation. Proceedings of the 27th Annual SAS® User Group International (SUGI’02).

[19] R Core Team (2014). R: A language and environment for statistical computing R Foundation for Statistical Computing, Vienna, Austria URL http://www.R-project.org/.

[20] Team R. RStudio: integrated development for R Boston, MA: RStudio. Inc. http://www.rstudio.com. 2015.

[21] Chang W, Cheng J, Allaire JJ, Xie Y, McPherson J. shiny: Web application framework for R. R package version 0.12. 2. 2015. 20.

[22] Holtkamp DJ, Polson DD, Torremorell M, Morrison B, Classen DM, Becton L et al Terminology for classifying swine herds by porcine reproductive and respiratory syndrome virus status. J Swine Health Prod. 2011; 19(1): 4. 22138772

[23] Dee SA. Elimination of porcine reproductive and respiratory syndrome virus from 30 farms by test and removal. J Swine Health Prod. 2004; 12(3):129–133.

[24] Linhares DC, Cano JP, Torremorell M, Morrison RB. Comparison of time to PRRSv-stability and production losses between two exposure programs to control PRRSv in sow herds. Prev Vet Med. 2014;116(1):111–9. 24931129 10.1016/j.prevetmed.2014.05.010

[25] Collins J, Dee S, Halbur P, Keffaber K, Lautner B, McCaw M, Yeske P. Laboratory diagnosis of porcine reproductive and respiratory syndrome (PRRS) virus infection. Swine health and production: the official journal of the American Association of Swine Practitioners (USA). 1996.

[26] Seo BJ, Kim HI, Cho HS, Park BY, Kim WI. Evaluation of two commercial PRRSV antibody ELISA kits with samples of known status and singleton reactors. J Vet Med Sci. 2016; 78(1):133–138. doi: 10.1292/jvms.15-0126 26290128 10.1292/jvms.15-0126 PMC4751132

[27] Rovira A, Clement T, Christopher-Hennings J, Thompson B, Engle M, Reicks D, MuZanzi C.Evaluation of the sensitivity of reverse-transcription polymerase chain reaction to detect porcine reproductive and respiratory syndrome virus on individual and pooled samples from boars. J Vet Diagn Invest. 2007; 19(5): 502–509. doi: 10.1177/104063870701900507 17823393 10.1177/104063870701900507

[28] Cameron A. Survey toolbox for aquatic animal diseases: a practical manual and software package. Monographs. 2002.

[29] Dean AG, Sullivan KM, Soe MM. OpenEpi: Open source epidemiologic statistics for public health, version. 2014.

